# Supplementary material for: MetaRibo-Seq measures translation in microbiomes
Source: Nat Commun. 2020 Jun 29;11:3268. doi: 10.1038/s41467-020-17081-z (PMC7324362; doi:10.1038/s41467-020-17081-z)
Supplement: Supplementary file 10 — Supplementary Data 7 [file 41467_2020_17081_MOESM10_ESM.zip › File2/Confidence_High_Taxonomy/156175_out.krona.html]

Javascript must be enabled to view this page.

members
magnitude
magnitudeUnassigned
count
unassigned
taxon
rank

156175\_out

115

115
superkingdom
2

phylum
976
115

200643
class
115

order
171549
115

114
family
815

36
816
114

SRS012849\_contig\_number\_35161SRS013476\_contig\_number\_contig-100\_131.220962SRS013800\_contig\_number\_20282SRS015579\_contig\_number\_25467SRS017103\_contig\_number\_35545SRS017191\_contig\_number\_contig-100\_2135.126452SRS017821\_contig\_number\_contig-100\_1397.192598SRS018936\_contig\_number\_9407SRS019381\_contig\_number\_6902SRS020328\_contig\_number\_contig-100\_30247.102167SRS023583\_contig\_number\_16302SRS024331\_contig\_number\_contig-100\_742.188083SRS043768\_contig\_number\_712SRS048870\_contig\_number\_contig-100\_2404.134526SRS049896\_contig\_number\_contig-100\_3821.186460SRS053356\_contig\_number\_contig-100\_1735.170389SRS053649\_contig\_number\_contig-100\_12277.87661SRS056259\_contig\_number\_29442SRS056273\_contig\_number\_contig-100\_769.153840SRS063985\_contig\_number\_32125SRS065504\_contig\_number\_contig-100\_4680.204353SRS075963\_contig\_number\_445SRS077392\_contig\_number\_43427SRS078419\_contig\_number\_contig-100\_1324.203849SRS1041037\_contig\_number\_contig-100\_6803.80575SRS1041136\_contig\_number\_contig-100\_11955.87799SRS140513\_contig\_number\_contig-100\_582.93180SRS144135\_contig\_number\_35296SRS145497\_contig\_number\_contig-100\_13639.83304SRS146812\_contig\_number\_58327SRS147653\_contig\_number\_contig-100\_1187.60354SRS148319\_contig\_number\_17312SRS893292\_contig\_number\_contig-100\_430.48853SRS893295\_contig\_number\_contig-100\_17142.56940SRS893300\_contig\_number\_11349SRS893342\_contig\_number\_9364
genus

246787
species

SRS024132\_contig\_number\_31979SRS063040\_contig\_number\_37
2

820
species
4

SRS022137\_contig\_number\_18787SRS055982\_contig\_number\_14661SRS065504\_contig\_number\_12544SRS101433\_contig\_number\_14018


SRS1041038\_contig\_number\_15049
1
2292929
species

species
818
12

SRS015264\_contig\_number\_17249SRS018133\_contig\_number\_contig-100\_884.83984SRS018984\_contig\_number\_30687SRS020233\_contig\_number\_contig-100\_1289.312846SRS020869\_contig\_number\_38313SRS045826\_contig\_number\_contig-100\_345.122803SRS048870\_contig\_number\_contig-100\_1349.124036SRS050299\_contig\_number\_14071SRS058070\_contig\_number\_contig-100\_811.164841SRS105153\_contig\_number\_38033SRS143181\_contig\_number\_14718SRS148721\_contig\_number\_contig-100\_1338.283510

species
1263042
1

SRS016018\_contig\_number\_contig-100\_377.71830

2

SRS024435\_contig\_number\_23489SRS058770\_contig\_number\_15381
species
46506

2292944
species

SRS045195\_contig\_number\_3049
1

12

SRS015960\_contig\_number\_19624SRS016381\_contig\_number\_358SRS017103\_contig\_number\_contig-100\_1101.208702SRS022071\_contig\_number\_32227SRS043667\_contig\_number\_5942SRS046717\_contig\_number\_2475SRS056519\_contig\_number\_11925SRS077086\_contig\_number\_13373SRS143523\_contig\_number\_22612SRS148511\_contig\_number\_18015SRS150029\_contig\_number\_20490SRS893230\_contig\_number\_11870
species
821


SRS148159\_contig\_number\_contig-100\_862.326738SRS149784\_contig\_number\_14679
2
species
47678

2292935
species
10

SRS014613\_contig\_number\_8262SRS017191\_contig\_number\_1387SRS020394\_contig\_number\_contig-100\_1459.95389SRS043701\_contig\_number\_17067SRS048060\_contig\_number\_12717SRS064232\_contig\_number\_4233SRS064276\_contig\_number\_5862SRS1054691\_contig\_number\_2907SRS1055043\_contig\_number\_18793SRS1055099\_contig\_number\_contig-100\_549.90771

469593
species

SRS148091\_contig\_number\_13316
1

30

SRS012273\_contig\_number\_47694SRS013098\_contig\_number\_43135SRS013940\_contig\_number\_19436SRS015890\_contig\_number\_7530SRS017701\_contig\_number\_21124SRS018049\_contig\_number\_833SRS018541\_contig\_number\_7319SRS022524\_contig\_number\_15325SRS022609\_contig\_number\_46459SRS023346\_contig\_number\_3210SRS043001\_contig\_number\_contig-100\_412.69089SRS048981\_contig\_number\_2438SRS052078\_contig\_number\_402SRS054590\_contig\_number\_1549SRS057717\_contig\_number\_2855SRS075341\_contig\_number\_22219SRS075878\_contig\_number\_23516SRS077024\_contig\_number\_2965SRS098655\_contig\_number\_11066SRS098717\_contig\_number\_14577SRS101268\_contig\_number\_22SRS1041112\_contig\_number\_1302SRS1041145\_contig\_number\_39295SRS1055034\_contig\_number\_9313SRS1055067\_contig\_number\_8821SRS140645\_contig\_number\_118SRS142712\_contig\_number\_4143SRS143598\_contig\_number\_2363SRS148874\_contig\_number\_4570SRS149879\_contig\_number\_26905
species
357276

1
2005525
family

1
375288
genus

species
46503

SRS018575\_contig\_number\_8131
1
